# The Investigation of Plasma Glucose-6-Phosphate Dehydrogenase, 6-Phoshogluconate Dehydrogenase, Glutathione Reductase in Premenauposal Patients with Iron Deficiency Anemia 

**Published:** 2014

**Authors:** Fatih Ozcicek, Mehmet Aktas, Kultigin Türkmen, T Abdulkadir Coban, Murat Cankaya

**Affiliations:** 1 Fatih Ozcicek, Department of Internal Medicine, Erzincan University, Medicine Faculty, Erzincan, 24100, Turkey.; 2 Mehmet Aktas, Biochemistry Department, Erzincan University, Medicine Faculty, Erzincan, 24100, Turkey.; 3 Kultigin Türkmen, Department of Internal Medicine and Nephrology, Erzincan University, Medicine Faculty, Erzincan, 24100, Turkey.; 4 T Abdulkadir Coban, Biochemistry Department, Erzincan University, Medicine Faculty, Erzincan, 24100, Turkey.; 5 Murat Cankaya, Science and Art Faculty, Biology Department, Erzincan University, Erzincan, Turkey.

**Keywords:** Anemia, Iron deficiency, glutathione reductase

## Abstract

***Background and Objective:*** Iron is an essential element that is necessary for all cells in the body. Iron deficiency anemia (IDA) is one of the most common nutritional disorders in both developed and developing countries. The glutathione pathway is paramount to antioxidant defense and glucose-6-phosphate dehydrogenase (G6PD)-deficient cells do not cope well with oxidative damage. The goal of this study was to check the activities of G6PD, 6-phosphogluconate dehydrogenase, glutathione reductase in patients with IDA.

***Methods:*** We analyzed the plasma samples of 102 premenopausal women with IDA and 88 healthy control subjects. Glucose-6-phosphate dehydrogenase and 6-phosphogluconate dehydrogenase activity as compared to the reduction of NADP +, glutathione reductase activity was performed based on the oxidation of NADPH. 2 ml of plasma were used in all analyzes. SPSS program was used for all of the statistical analysis.

***Result:*** Diagnosis of iron deficiency in patients belonging to the analysis of blood were ferritin 3.60 ± 2.7 ng / mL, hemoglobin 9.4 ± 1.5 mg / dl and hematocrit 30.7 ± 4.1% ratio; in healthy subjects ferritin 53.5 ± 41.7 ng/ml, hemoglobin level 13.9 ± 1.3 mg / dl and hematocrit ratio 42 ± 3.53%. When compared to healthy subjects the glutathione reductase level (P<0.001) was found to be significantly higher in patients with IDA. IDA patients with moderate and severe anemia had lower GR activity when compared to IDA patients with mild anemia. But the plasma levels of glucose-6-phosphate dehydrogenase (P<0,600) and 6-phosphogluconate dehydrogenase (P<0,671) did not show any differences between healthy subjects and in patients with IDA.

***Conclusion:*** It was shown that Glucose-6-Phosphate Dehydrogenase and 6-Phosphogluconate Dehydrogenase have no effect on iron-deficiency anemia in patients. The plasma GR levels of premenopausal women with IDA were found to be higher compared to healthy subjects, which could be secondary to erythrocyte protection against oxidative stress being commonly seen in IDA.

## INTRODUCTION

Iron deficiency anemia (IDA) is one of the most common nutritional disorders in both developed and developing countries.^1^ The prevalence of anemia was found to be 6% and 27% in developed and developing countries, respectively.^2^ In Turkey, Isik et al.^3^ found that the overall prevalence of anemia was 5.6% in adolescents. Of these patients, 59% were diagnosed as IDA.^3^ Glucose-6-phosphate dehydrogenase (G6PD) catalyzes the rate-determining step in the pentose phosphate pathway and produces NADPH to fuel glutathione recycling. G6PD deficiency is the most common enzyme deficiency in humans and affects over 400 million people worldwide.^4^ The glutathione pathway is paramount to antioxidant defense and G6PD-deficient cells do not cope well with oxidative damage.^5^ Normal values of G6PD, 6-phosphogluconate dehydrogenase (6PGD) and glutathione reductase (GR) have been determined in normocytes, reticulocytes, newborn cord erythrocytes and leukocytes.^6^

Iron is a vital mineral that is widely encountered in biological systems. Organisms contain one of two oxidative forms of iron such as ferrous state (Fe^2+^) and ferric state (Fe^3+^).^7^ Serum levels of iron are dependent on age, gender, race, geographic region, and socio-economic status.^8^ According to World Health Organization (WHO), one of the most common nutritional disorders is iron deficiency anemia in both developed and developing countries.^9^^,^^10^ In addition, WHO estimated that IDA caused approximately 273,000 deaths/year, of which the 97% occurred in low- and middle-income countries.^11^

In addition to its traditional roles in glycolysis and antioxidant cellular defense systems, the pentose phosphate pathway (PPP) was found to be associated with angiogenesis, cell survival and apoptosis.^12^ One of the most important salvage pathway that supplies ribose 5-phophate, NADPH, other glycolytic intermediates and end-products of cells is PPP.^4^ G6PD and 6PGD are the key enzymes that provide NADPH (the reduced form of the NADP) by converting glucose-6-phosphate to 6-phospho-D-gluconolactone and 6-phospho D-gluconolactone to D-ribulose 5-phosphate, respectively.^5^ Cells use this NADPH to preserve glutathione in its reduced form as GSH.^13^ The other enzyme named glutathione reductase is also essential to convert GSSG to GSH.^14^ PPP is the unique way to produce GSH especially in cells without mitochondria. In their lifespan, erythrocytes are prone to oxidative stress. Hence, the fate of the erythrocytes depends on the ability to fight against oxidative stress which is also highly reliant to G6PD, 6PGD and GR enzyme activities.^6^

To date in the literature, the data regarding metabolic enzymes mentioned above and iron deficiency is scanty. Hence we aimed to investigate the relationship between iron deficiency and glucose-6-phosphate dehydrogenase, 6-phosphogluconate dehydrogenase and glutathion reductase enzyme activities in patients with IDA.

## METHODS

The study protocol was approved by the Medical Ethics Committee of Erzincan University (Mengucek Gazi Training and Research Hospital School, Erzincan, Turkey). Written informed consent was obtained from all participants.

Between February and June 2012, this cross-sectional study was performed in 102 premenopausal women with IDA **(**mean age: 39.3±12.1 years) and 88 healthy control subjects (premenopausal women, mean age: 35.1±13.1 years).

We included in this study the patients (age range from 18 to 55 years) demanding the assessment of iron deficiency anemia. A review of medical records was used to assess patients for study enrollment. To diagnose IDA, the concentration of ferritin <15 ng/mL was accepted as cut-off.^15^ Exclusion criteria included anemia other than iron deficiency, pregnancy, blood-transfused patients in the last 6 months, documented coronary artery disease, congestive heart failure, active and chronic infection, and autoimmune disease. Of 145 patients, 49 were excluded from the study, including 25 (macrocytic anemia), 4 (pregnancy), 3 (blood transfusion in the last 6 months), 3 (congestive heart failure) (New York Heart Association class III–IV), 5 (active infection), 2 (chronic infection), and 1 (an autoimmune disease). The remaining 102 premenopausal women with IDA were included in the study. Healthy age- and sex-matched premenopausal individuals referred from outpatient clinics of the Internal Medicine Department of Erzincan University (*n* = 88) were taken as control subjects. They had to meet the same inclusion and exclusion criteria as the patients.

Patients were divided in three groups according to WHO, which defines mild anemia as Hb 11.0-11.9 g/dl, moderate as Hb 8.0-10.9 g/dl and Hb <8.0 g/dl.^16^

***Chemicals: ***6PG, G6P, GSSG, NADPH, EDTA and NADP^+^ were obtained from Sigma-Aldrich Co. All other chemicals were analytical grade and obtained from Merck.

***Preparation of the Hemolysate: ***Erythrocytes were purified from fresh human blood obtained from the Blood Centre of Mengucek Gazi Training and Research Hospital School. The plasma samples were stored at−40^o^C until analysis. The blood samples (2 ml) were centrifuged at 2,250xg for 15 min, and the serum and buffy coat were removed. The packed red cells were washed three times with KCl (0.16 M) and hemolyzed with equal volume of ice-cold water and then centrifuged (4^o^C, 10,000xg, for 30 min) to remove the ghosts and intact cells.^17^

***Measurement of glutathione reductase (GR) activity: ***Beutler’s method was slightly modified for measurement enzymatic activity at 25^o^C.^14^^,^^18^ One enzyme unit is defined as the oxidation of 1 µmol NADPH per min under the assay conditions at 25^o^C.

***Measurement of glucose-6-phosphate dehydrogenase (G6PD) activity: ***G6PD was measured spectrophotometrically at 25^o^C as described by Beutler.^18^ The activity measurement was done by monitoring the increase in absorption at 340nm due to the reduction of NADP^+^ at 25^o^C. One EU was defined as the enzyme reducing 1mmol NADP^+^ per min at 25^o^C and optimal pH (pH 8.0).

***Measurement of 6-phosphogluconate dehydrogenase (6PGD) activity: ***The enzymatic activity of 6PGD was measured by Beutler’s method.^18^ NADPH produced in the reaction mixture was measured at 340 nm. One unit of enzyme (EU) activity was defined as the enzyme amount reducing 1 mol NADP+ per min at 25°C, pH 8.0.

***Statistical Analysis: ***Statistical analyses were performed using the Statistical Package for Social Sciences (Windows version 19.0: SPSS, Chicago, IL, USA). Data were reported as mean± standard deviation. The normal distribution of all variables was tested using the Kolmogorov–Smirnov test. Between-group statistical differences for parametric data were analyzed using the Student t-test. For nonparametric data, the Mann–Whitney U-test and Kruskal-Wallis test were used. *p* < 0.05 was considered significant for all tests.

***Biochemical analyses: ***Venous blood samples for biochemical analyses were drawn from 102 patients with IDA and in 88 healthy subjects. All biochemical analyses including those for hemogram, hematocrit and serum ferritin levels were undertaken using an oxidase-based technique by the Roche/Hitachi Modular System (Mannheim, Germany) in the Central Biochemistry Laboratory of the Erzincan University Mengucek Gazi Training and Research Hospital.

## RESULTS

***Baseline characteristics of patients and healthy subjects: ***The baseline characteristics of 102 patients with IDA and 88 healthy subjects are shown in Table-I. There were no differences in age between patients and healthy subjects. However, patients with IDA had significantly lower hemoglobin, hematocrit and serum ferritin levels (Table-I).

We compared the activity of enzymes in question between patient and the healthy groups (Table-II). The plasma levels of glutathione reductase were found to be significantly higher in patients with IDA compared to the healthy subjects (3.16±3.34 and 1.11±3.05, respectively) (p<0.001). On the other hand, there were no statistically significant difference between patient group and healthy subjects in terms of plasma G6PD and 6PGD (Table-II). IDA patients with moderate and severe anemia had lower GR activity when compared to IDA patients with mild anemia. Our Enzymatic and non-enzymatic parameters of all the four groups summarized in Table III.

## DISCUSSION

The main findings of the present study were as follows: i) plasma glutathione peroxidase levels were found to be higher in patients with IDA when compared to healthy subjects. ii) In contrast, there were no statistically significant difference between patients with IDA and healthy subjects in terms of plasma G6PD and 6PGD activities. iii) IDA patients with moderate and severe anemia had lower GR activity when compared to IDA patients with mild anemia.

**Table-I:** Demographic, clinic and laboratory features of patients and control groups

***Parameters***	***Patients with IDA (n=102)***	***Healthy Subjects (n=88)***	***P value*** *
Age (years)	39.3±12.1	35.1±13.1	0.083
Hemoglobin (mg/dL)	9.4±1.5	13.9±1.3	**<0.001**
Hematocrit (%)	30.7±4.1	42±3.53	**<0.001**
Ferritin (ng/ml)	3.60±2.7	53.5±41.7	**<0.001**

* *Student t-test*

**Table-II:** The plasma levels of GR, G6PD, 6PGD of patients with IDA and in healthy subjects

***Parameters***	***Patients with IDA (n=102)***	***Healthy Subjects (n=88)***	***P value*** *
Glutathion Reductase	3.16±3.34	1.11±3.05	**<0.001**
Glucose-6-phosphate dehydrogenase	2.26±2.63	1.89±2.76	0,600
6-phosphogluconate dehydrogenase	3.73±4.08	3.13±5.27	0,671

* *Mann–Whitney U-test.*

**Table-III:** The levels of GR, G6PD, 6PGD of patients according to anemia groups

***Parameters***	***Healthy subjects (n=88)***	***Mild Anemia*** ***(n=20)***	***Moderate Anemia)*** ***(n=63)***	***Severe Anemia*** ***(n=19)***	***P value*** *
Hb	13.9±1.3	11.3±0.3	9.6±0.9	7.1±0.8	**<0.001**
Hct	42±3.5	35.5±2.0	31.1±2.7	24.9±2.6	**<0.001**
Ferritin	53.5±41.7	4.9±3.1	3.5±2.5	2.7±2.4	**0.039**
Glutathion Reductase	1.11±3.05	4.73±3.98	2.82±3.06	2.95±3.52	**<0.001**
Glucose-6-phosphate dehydrogenase	1.89±2.76	3.52±2.97	2.14±2.70	1.50±1.41	0.083
6-phosphogluconate dehydrogenase	3.13±5.27	4.23±4.37	3.32±3.94	4.87±4.36	0.443

* *Kruskal-Wallis Test.*

To our knowledge, this is the first study evaluating the plasma levels of GR, G6PD and 6PGD activities in patients with IDA.

Glutathione reductase catalyzes glutathione (GSH) to glutathione disulfide (GSSH) by using NADPH. GSH also plays an important role in the conversion of Fe^+2 ^to Fe^+3^.^19^ In the present study, we found that plasma GR enzyme activity is increased in IDA patients compared to healthy subjects. Additionally, we also demonstrated that IDA patients with moderate and severe anemia had lower GR activity when compared to patients with mild anemia. In accordance to our results, Tiwari et al.^20^ showed that plasma GR levels were lower in severe anemia and higher in mild anemia in pregnant women with IDA. This may be secondary to protective effects against oxidative stress of erythrocytes via its role in converting GSSG to GSH.^21^

In a rat model, Dhur et al.^22^ demonstrated that severe and moderate iron deprivation might result in a stimulation of G6PD and 6PGD activities per million erythrocytes and even moderate iron deficiency may alter fundamental enzymatic systems intervening in drug metabolism and in the pentose phosphate pathway. However, we could not find any statistical differences between IDA patients with mild, moderate and severe anemia and healthy subjects regarding plasma G6PD and 6PGD activities in our study.

Our study has two main limitations. First, the analysis of patients with IDA focusing in the plasma levels of GR, G6PD and 6PGGH was cross-sectional in nature. Secondly, as mentioned earlier, the sample number was relatively small. Being that this was not a prospective controlled study, we cannot draw cause-and-effect conclusions from our findings.

In conclusion, plasma GR levels of premenopausal women with IDA were found to be higher compared to healthy subjects, which could be secondary to erythrocyte protection against oxidative stress being commonly seen in IDA. There are many missing pieces in the puzzle. Hence, in-vitro and experimental studies are needed.
